# Assessing the impact of the Australia-United States Free Trade Agreement on Australian and global medicines policy

**DOI:** 10.1186/1744-8603-1-15

**Published:** 2005-10-06

**Authors:** Thomas Faunce, Evan Doran, David Henry, Peter Drahos, Andrew Searles, Brita Pekarsky, Warwick Neville

**Affiliations:** 1 Globalisation and Health Project, Centre for Governance of Knowledge and Development Regulatory Institutions Network Australian National University, Acton, Canberra ACT, Australia; 2 Medical School and Law Faculty, The Australian National University, Acton, Canberra, ACT Australia; 3 Newcastle Institute of Public Health, University of Newcastle, Newcastle, New South Wales, Australia; 4 Clinical Pharmacology, School of Medical Practice and Population Health, University of Newcastle, Newcastle, New South Wales, Australia; 5 Centre for Regulation and Market Analysis, University of South Australia, Adelaide, South Australia, Australia

## Abstract

On 1 January 2005, a controversial trade agreement entered into force between Australia and the United States. Though heralded by the parties as facilitating the removal of barriers to free trade (in ways not achievable in multilateral fora), it also contained many trade-restricting intellectual property provisions and others uniquely related to altering pharmaceutical regulation and public health policy in Australia. The latter appear to have particularly focused on the world-respected process of federal government reimbursement after expert cost-effectiveness evaluation, popularly known as the Pharmaceutical Benefits Scheme ('PBS'). It remains uncertain what sort of impacts – if any – the Australia-United States Free Trade Agreement ('AUSFTA') will have on PBS processes such as reference pricing and their important role in facilitating equitable and affordable access to essential medicines.

This is now the field of inquiry for a major three year Australian Research Council ('ARC')-funded study bringing together a team of senior researchers in regulatory theory from the Australian National University and pharmacoeconomics from the University of Newcastle. The project proposes to monitor, assess and analyse the real and potential impacts of the AUSFTA in this area, providing Australian policy-makers with continuing expertise and options.

To the extent that the AUSFTA medicines provisions may represent an important precedent in a global strategy by industry on cost-effectiveness evaluation of pharmaceuticals, the study will also be of great interest to policy makers in other jurisdictions.

## Introduction

The final text of the Australia-United States Free Trade Agreement ('AUSFTA') was signed in Washington on 18 May 2004, by the Australian Trade Minister and the United States Trade Representative. On 17 November 2004, the parties exchanged notes accepting their respective implementing processes and the agreement entered into force on 1 January 2005. The AUSFTA contained numerous provisions either directly or indirectly related to medicines regulation in Australia, particularly Annex 2C of Chapter Two, Chapter Seventeen on intellectual property and Chapter Twenty One on dispute resolution.

It remains uncertain whether the AUSFTA will have either a detrimental or beneficial impact on access to medicines and the promotion and maintenance of good health in Australia. There does, however, appear to have been a substantial difference in opinion between the Parties over procedural changes that would result in Australian medicines regulation.

Throughout the negotiations, the Australian Government's position was either that the government cost-effectiveness reimbursement system, the Pharmaceutical Benefits Scheme ('PBS'), would not be included in the AUSFTA, or that if it was, it was an item of public health policy whose core components would be protected[1]. After signature, the Australian government maintained that the fundamental architecture of the PBS remained unchanged. It acknowledged commitments to make improvements to the transparency and timeliness of PBS processes. It also affirmed its reasonable expectations that, as a result of the AUSFTA, Australian citizens would benefit from faster access to new prescription medicines, that the price of medicines on the PBS would not increase and that the text of the AUSFTA made no changes to the cost-effectiveness methods used to set PBS reimbursement levels[2].

On the other hand, the Deputy US Trade Representative stated to the US Congress:

*The U.S.-Australia FTA is the first to include non-tariff market access provisions to address issues in the pharmaceutical sector. Recognizing the sensitivity of this issue, we drew on studies prepared by the Australian government to propose changes that would improve transparency and the regulatory procedures for listing new drugs in Australia. Under the FTA, the United States and Australia agreed to common principles on facilitating high quality health care and continued improvements in public health, including through government support for research and development in the pharmaceutical industry. We also agreed to establish a Medicines Working Group to discuss emerging health policy issues. Australia committed to specific steps to improve the transparency, accountability and promptness of the listing process, including establishment of an independent review of listing decisions*[3].

Representatives of the multinational brand-name pharmaceutical industry, including its regional organisation Medicines Australia, claimed that there was no basis to claims that the US wanted the PBS dismantled[4]. They argued that the regulatory changes required by these areas of the AUSFTA would (a) help redress an alleged current undervaluing of pharmaceutical 'innovation' in Australian pricing arrangements and (b) stimulate locally-based research and development, as well as the local, mostly generic, pharmaceutical industry[5]. They asserted the negotiated modifications would make Australia's regulatory system more oriented to the global market pressures on industry, more responsible in its approach to intellectual property rights and so more attractive to private investment, resulting in a net welfare benefit[6].

Others, however, have pointed to US legislation requiring that nation's negotiators to seek in the AUSFTA provisions facilitating the "elimination of government measures such as price controls and reference pricing which deny full market access for United States [pharmaceutical] products"[7]. The Australian Senate Select Committee on the AUSFTA concluded:

*While no single one of the specific commitments will create immediate and measurable price rises for the PBS, the new measures may well over time alter the bargaining power between the PBS and pharmaceutical companies. This may have long term ramifications that are not in the interest of Australian consumers*[8].

Concern has been expressed about AUSFTA provisions with the potential to encourage higher medicines prices in Australia. These include provisions in chapter 17 (Intellectual Property) that expand the obligations of the Trade Related Intellectual Property Rights ('TRIPS') agreement by prohibiting parallel importation, restricting compulsory licensing to "national emergencies of extreme urgency," prohibiting generic manufacturers exporting to a patent-expired market when a domestic patent exists and increasing data exclusivity protections[9].

A significant additional worry for these commentators was article 17.10.4. For the first time in Australia, this linked generic regulatory market approval on quality and safety grounds with the patent status of the relevant brand name product[10]. This Hatch-Waxman-type provision was felt to risk brand name manufacturers "evergreening" soon-to-expire pharmaceutical patents, as had occurred after comparable regulations were introduced in jurisdictions such as the US and Canada[11]. The academic, community and parliamentary concern in Australia was so great on this issue, that it resulted in the Australian government passing "anti-evergreening" amendments to its AUSFTA implementing legislation. These imposed a $A10 million penalty for a bad faith challenge by a brand name manufacturer of a generic notification certificate under the new s26B of the *Therapeutic Goods Act 1989 (Cth). *They also allowed cost recovery in such circumstances by the Australian government[12].

Provisions in Annex 2C(1) emphasising the need for increased government recognition of pharmaceutical "innovation" and "research and development" were likewise viewed by such critics as having the potential to encourage brand name industry lobbying. This could potentially weaken, in the long term, the capacity of Australia's Pharmaceutical Benefits Advisory Committee ('PBAC') to reject, on clinical and cost effectiveness grounds, new medicines from inclusion in the government's PBS positive reimbursement list, or to reference their reimbursement price against older products with equivalent efficacy but much reduced price[13].

In this paper, we present a rationale and outline a draft plan for a three-year study, funded by the Australian Research Council ('ARC'), which will examine the impact of the AUSFTA on a range of regulatory, public health and industrial interests involved with access to medicines in Australia. An important initial point to make is that we consider the AUSFTA is best researched as a component of an ongoing process of interaction with Australia's medicines policy by the global pharmaceutical industry. This trade agreement should be viewed, in other words, either as a catalyst that may enhance the speed of regulatory change, or a tangible manifestation of industry lobbying principles that, till now, may have been more implicit. It would be misleading, in any event, to investigate the AUSFTA's potential impacts on Australian medicines policy in isolation of demonstrable long-term corporate strategies.

Some central issues our study will examine include to what extent the AUSFTA requires, facilitates, or is likely to result in, changes to Australia's generic pharmaceutical industry, as well as its PBS cost-effectiveness system of pharmaceutical regulation. We also aim to consider relevant net welfare gain or loss; whether the Australian community will get the same value-for-dollar spent on medicines, either through Commonwealth government reimbursement, hospital or patient purchase.

We propose to investigate these questions empirically (and provide a sound structure for the gradual acquisition of suitable data). This will be done first by identifying, with the assistance of qualitative interviews, actual or likely AUSFTA-associated changes to the structure and process of Australia's PBAC, as well as the marketing processes, development and sector competitiveness of generic pharmaceutical manufacturers in Australia. This aspect of the study will also review the legitimacy of such actual or proposed alterations by examining the history of Australia's PBS as a social justice measure designed to ensure universal access to essential medicines. We will also review such proposed changes for coherence with basic norms of bioethics, domestic law and international human rights. We shall then attempt to determine their actual or potential impact on a range of indicators including drug prices expenditure and affordability, drug availability and equity of access.

We hope that publishing an outline of our proposed study will further encourage policy discussion, facilitate collaborations and provide a template for governments of other countries planning to enter such agreements. Although much of the detail of the AUSFTA is specific to Australia, there are important elements likely to be relevant to future trade agreements involving the US or other countries that have a major vested interest in the production, export and rent generation associated with patented medicines. These include whether the strengthening of pharmaceutical intellectual property protection and weakening of medicines clinical and cost-effectiveness evaluation and/or reference pricing, necessarily involves a weakening of a nation's social and economic fabric, or the capacity of its population to age well and age productively.

## Background: Australia's PBS in the Context of the AUSFTA

Australia's pharmaceutical sector is dominated by the operation of the federally funded PBS, which, after a process of clinical and cost-effectiveness evaluation contributing recommendations to price negotiation, provides reimbursement (currently approximately 75%) for around 80% of the prescription medicines used in Australia[15]. The PBS does not restrict market access, but facilitates maximisation of sales volume for listed products. In developing relevant price indices, our study will also take into account AUSFTA impacts on prices for hospital-used medicines (which can be calculated from the PBS reimbursement price less the minimum safety net value) and predict expenditure on medicines costed under the co-payment level.

Central to our analysis of the impact of the AUSFTA on medicines in Australia, however, will be an evaluation of its effect on the PBS. Australia's PBS was established as a free formulary of essential drugs after the Second World War by the Curtin-Chifley federal administrations[16]. It was a social justice measure designed to ensure that all Australian citizens gained access to affordable, essential medicines. Legislation to create the PBS had to survive two High Court challenges and required a successful Constitutional referendum[17]. Successive Commonwealth governments used and built upon the 1940s enactments, before a conservative party enacted the *National Health Act 1953 *(Cth) ('*National Health Act')*[18]. This is an extremely important point, that will be focused on by ARC research scholar Warwick Neville. The PBS is one of the few examples of public health policy in Australia's history that appears to have an unequivocal democratic mandate. Such an historical-jurisprudential perspective on the social justice aspects of the PBS will be a unique and distinctive feature of our analysis.

The modern PBS revolves around Part VII section 85 of the *National Health Act 1953 (Cth)*. Section 101(4) of this Act states that the relevant Minister, upon the advice of the PBAC (with secretariat support from the Pharmaceutical Benefits Branch of the Department of Health and Ageing ('DOHA'), may declare a pharmaceutical listed on the PBS and so subject to a level of government reimbursement (except for a patient co-payment) that has been negotiated by the Pharmaceutical Benefits Pricing Authority ('PBPA'). Prior to the AUSFTA, PBS listing was required to only occur after the Therapeutic Goods Administration ('TGA') and the Australian Drug Evaluation Committee ('ADEC') had approved the relevant pharmaceutical's safety and efficacy[19]. The AUSFTA, through implementing amendments to the *Therapeutic Goods Act 1989 (Cth)*, has already produced, as mentioned, a requirement that a market-entering generic manufacturer provide evidence that no counteracting patent is claimed, or that the brand name owner has been notified.

Central to the PBAC's clinical and cost-effectiveness evaluation is section 101(3) of the *National Health Act*. This requires the PBAC to base its recommendation on: 'the effectiveness and cost of therapy involving the use of the drug, preparation or class, including by ***comparing the effectiveness and cost ***[emphasis added] of that therapy with that of alternative therapies, whether or not involving the use of other drugs or preparations.' The section goes on to state that if the product is 'substantially more costly' than the selected comparator in its class it shall not be recommended by the PBAC for PBS listing 'unless...[it] provides a significant improvement in efficacy or reduction of toxicity over the alternative therapy or therapies.'

The PBS system that has evolved under this section is a variant of pharmaceutical reference pricing[20]. The pharmaceutical manufacturer's ('sponsor's') submission to the PBAC nominates a disease indication (and relevant subsets involving patient characteristics) as well as a listing price supposedly based on the pharmaceutical company's assessment of the best relevant available data on clinical effect against a comparator. The comparator is generally the drug most prescribed on the PBS for the same indication, but may be the standard medical (non-drug) treatment. Pharmaceutical companies tend to prefer comparisons against the most expensive drug with the best 'head-to-head' data, rather than the compound that is most pharmacologically similar[21]. Part of our initial task will be to determine what aspects, if any, of this important public health-related process could be subjected to pressure emerging from the AUSFTA. It will be an interesting threshold question to ascertain to what extent members of the PBAC were aware of, or consulted in, the development of the AUSFTA articles relevant to the PBS.

The PBAC's expert reviewers evaluate whether any of the assumptions in the submission are unjustifiable and create simulations to assess the incremental cost-effective ratio (the additional cost for an additional beneficial effect, or Quality of Life Years ('QALY') gained)[22]. The reports of these experts ('pink pages') are then passed back to be reviewed by the PBAC, along with an industry response to them ('blue pages') and the summary from the Economic Sub-Committee ('ESC') in the 'green pages.' The process is designed to take six weeks and follows guidelines set out on the PBS website. The extent to which such guidelines alter as a result of the AUSFTA and what impact this has on the PBAC process will be another aspect of our study.

The PBAC may ask the pharmaceutical manufacturer for additional information, but has no legal power to compel its production, even if not covered by 'commercial-in-confidence' protections. The pharmaceutical manufacturer may be claiming a price premium because of a claimed additional benefit (that is improved effectiveness, better adverse event profile or delivery system) conferred by the new product over its therapeutic rivals. One hypothesis we hope to test is that brand-name pharmaceutical manufacturers may use Annex 2C(1) of the AUSFTA to seek price premiums for alleged "innovation" as a separate issue from cost-effectiveness. If the relevant evidence submitted does not clearly establish clinical and cost effectiveness, then a process of cost minimization is undertaken[23]. The extent to which members of the PBAC are aware of any broader, strategic agenda of the brand name pharmaceutical industry in making individual submissions (for example on reference pricing), will also be an aspect of our research in this area.

Once a decision is made to list the drug, the PBPA then evaluates the requested price against an international benchmark price for drugs in that class. Thus, under the PBS system, the members of the PBAC and ESC use pharmacoeconomic analysis to determine the community value of a new drug against an agreed comparator therapy, while the monopsony bargaining power of the PBPA is used to counter the increasingly prolonged and wide potential for monopoly rents accorded to brand name pharmaceutical patent holders[24]. For brand name manufacturers, the process of bringing a new patented product to the Australian market at a higher price than currently available medicines may be lengthy, expensive and uncertain. However PBS listing provides a secure foothold in a substantial market, particularly (due to reference pricing) for the generic pharmaceutical industry[25].

The predominant norms underlying the current Australian system are cost-containment, efficiency and equity. When evaluated against such standards the PBS has performed well[26]. The considerable monopsony bargaining power it offers to government has resulted in lower overall prices for the medicines listed on the PBS. Lacking a capped budget and dependent upon prescribers following the approved guidelines, the PBS process, however, was viewed, prior to the AUSFTA, as primarily involved with quality use of medicines, rather than government cost containment[27].

Brand-name manufacturers have argued that PBS-type systems involving reference pricing, create a regulatory environment hostile to investment and innovation. Specifically, they claim, as they have in other jurisdictions, that reference pricing makes it progressively more difficult for innovative brand-name pharmaceuticals to enter the market at a price sufficient to recoup the cost of research and development. The low prices achieved through such tactics have also been alleged by such manufacturers to reduce the potential for locally based industry expansion and to risk eventual precipitation of a withdrawal of international manufacturers from the sector. They claim that Australia can only achieve the low price it currently commands for innovation by opportunistically "free-riding" on the research and development spending of developed nations, such as the US. The US Department of Commerce has recently produced a report criticising medicines price controls in OECD countries, which applies the same arguments to those jurisdictions[28]. These claims will need to be tested against available evidence.

## Medicines-Related Provisions of the AUSFTA

The AUSFTA is one of a number of recent bilateral agreements sought by the US in its strategy of negotiating stronger intellectual property rights ('IPRs') with smaller trading partners. So-called "TRIPS-Plus" IPRs, seen by the US as essential for the protection of the monopoly rent it draws from intangible assets such as pharmaceutical patents, are an important feature of the AUSFTA. The AUSFTA medicines provisions also arise from an intention on the part of the US pharmaceutical industry, through the USTR, to modify government evaluative structures and processes to increase communication between industry and regulators while strengthening the financing of innovation through research and development. The extent to which this aim succeeded is debateable.

The AUSFTA, to summarise, contains approximately fifty provisions in four areas relevant to Australia's pharmaceutical sector: Annex 2C (Pharmaceuticals); the side-letters between the Australian Trade Minister and US Trade Ambassador; Chapter 17 (Intellectual Property Rights); and Chapter 21 (Dispute Resolution Procedures)[29].

Annex 2C(1) begins by articulating one overarching principle – that the parties to the agreement are '...committed to facilitating high quality health care and continued improvements in public health for their nationals.' It then mentions four subsidiary principles:

a) [recognising] the important role played by innovative pharmaceutical products in delivering high quality health care;

b) [recognising] the importance of research and development in the pharmaceutical industry and of appropriate government support, including through intellectual property protection and other policies;

c) the need to promote timely and affordable access to innovative pharmaceuticals through transparent, expeditious, and accountable procedures, without impeding a Party's ability to apply appropriate standards of quality, safety, and efficacy; and

d) the need to recognize the value of innovative pharmaceuticals through the operation of competitive markets or by adopting or maintaining procedures that appropriately value the objectively demonstrated therapeutic significance of a pharmaceutical.

Other subsections of Annex 2C, and the associated exchange of letters, relate to increased opportunities for a manufacturer to interact with regulators. This includes Australia providing manufacturers with an opportunity for hearings before the PBAC during the application for PBS listing; providing an opportunity for an independent review process following a negative PBAC price determination; the creation of a Medicines Working Group with health officials from each country in dialogue about aspects of Australia's regulatory mechanisms; an ongoing dialogue between the TGA and the US Food and Drug Administration on the issue of making pharmaceutical innovation 'quickly available'; and finally, Annex 2C(5) permits a pharmaceutical manufacturer to disseminate information about pharmaceutical innovation via the Internet.

Chapter 17 of the AUSFTA includes intellectual property provisions aimed specifically at Australia's pharmaceutical sector. Parallel importation, as already mentioned, is prohibited; compulsory licensing of pharmaceuticals is restricted to a standard more stringent than that applying in TRIPS ("national emergencies of extreme urgency"); generic production of domestic-patented drugs for export to jurisdictions where patents have already expired is prevented. Data exclusivity is extended, as is patent protection where there have been delays in issuing marketing approval[30]. Article 17.10.4 of the AUSFTA requires the Australian TGA to "prevent" marketing approval for a generic product whenever any type of patent is "claimed" for brand-named drug. Australian implementing legislation, however, while creating the required notification process, has imposed penalties for "evergreening" which resemble similar provisions in both the US and Canada[11].

Under the dispute resolution Chapter 21, a panel of three nominated trade lawyers will have the power to interpret compliance with obligations in the AUSFTA. Article 21.2(c) contains what is known in international trade law as a non-violation nullification of benefits ('NVNB') article[31]. Such articles allow dispute resolution proceedings to be commenced where only the spirit of the treaty had been broken, or more technically, legitimate expectations have been nullified [32]. Australia may be able to use this provision to argue that its legitimate expectation was that the AUSFTA would lead to no amendment of the *National Health Act 1953 (Cth) *and in particular to the mechanism therein of cost-effectiveness pricing of pharmaceuticals.

## Potential impacts of the AUSFTA on Medicines in Australia

On the face of it, and as argued by the Australian government, the provisions of the AUSFTA represent procedural changes rather than substantive reform to current regulatory arrangements. In any event, there negotiations have seen principles such as recognition of pharmaceutical innovation set here in a unique public health context. Within the principles of Annex 2C(1), for example, 'innovation' is linked with high quality health care, 'affordability', 'accountability' and 'objectively demonstrated therapeutic significance'. This linkage is arguably reflective of the current Australian approach of defining innovation with regard to its comparative therapeutic value, that is its clinical and cost-effectiveness. Further, Annex 2C(1) commits both parties to promoting 'affordable' access to innovative drugs and to a recognition of innovation that may involve either competitive markets (that is, a market not dominated by monopolistic patents) or procedures that appropriately value the objectively demonstrated therapeutic significance of a pharmaceutical (such as, but not specifically referring to, the system under Australia's PBS).

However, the interpretive principles of Annex 2C(1), do not specifically refer to the PBS (unlike those in Annex 2C(2) on transparency). They are also sufficiently vague to allow considerable scope in interpreting what obligations they create. Despite its apparent centrality to the lobbying agenda of the brand-name pharmaceutical industry, 'innovative' is not defined explicitly (here or anywhere else in the text of the AUSFTA) and the precise obligations created by a requirement for stronger recognition of pharmaceutical 'innovation' are not clear. This possibly deliberate lack of clarity extends to other provisions of Annex 2C, such as the creation on an 'independent review process' and the 'Medicines Working Group'. The crucial concept of transparency, is also not unambiguously defined in the AUSFTA.

The problem, which should not be understated, is that where such differences in interpretation are structured into the agreement, disputes about expectations and obligations are only postponed, rather than resolved[33]. It may be important to consider, therefore, the effect of Annex 2C(1) framing such obligations on governments to recognise pharmaceutical "innovation" and the research and development necessary for it within the overarching obligation of industry to objectively prove the contribution of such products to overall public health. It could also be relevant to study whether these changes facilitate policy proposals with the capacity to diminish equity of access to essential medicines (contrary to the National Medicines Policy), leading to reduced health outcomes for elderly citizens and those reliant on such therapies for quality of life and productivity. Such proposals could include patient co-payment rises, means-tested co-payments, medicines savings accounts, changes to reference pricing, to the pricing of generic pharmaceuticals, or a diminution of the capacity of the PBAC to make cost-effectiveness recommendations[14].

If the principles and provisions of Annex 2C represent the 'spirit' of the AUSFTA regarding pharmaceuticals – it will be important to research to what extent that Australia could satisfactorily meet that spirit and continue to apply pharmaceutical reference pricing, or prohibit direct-to-consumer advertising. Policy suggestions that we could research here include the creation of a pharmaceutical "innovation" prize system outside the PBS.

The provisions in each area of the AUSFTA articulate with the provisions in other areas. Should the US determine that the spirit of Annex 2C is not being met, it is highly plausible that it could seek redress by invoking the NVNB clause in Chapter 21. In this context, an important component of our research will be to examine whether the new ss26C and D of the *Therapeutic Goods Act 1989 (Cth) *are a "dead-letter," as some have suggested (due to reasonable exceptions grounds and the uncertain incentives for Australian generic manufacturers to bring such actions), or whether these amendments may play an important role in clarifying Australia's legitimate expectations in this area, for the purposes of a subsequent NVNB trade dispute action under article 21.2(c).

The overall significance here is that future PBAC decisions not to list 'innovative' new drugs from US companies (because they were judged not cost-effective) will be made in the shadow of possible US trade retaliation in important areas such as manufacturing and agriculture[34]. What effect such a shadow might have on the deliberative processes of Australian regulators is difficult to predict and indicators of such pressure may need to be established.

Thus, while this version of the AUSFTA (a supervising committee under chapter 21 may recommend changes) does not ostensibly seek to modify the basic architecture of the PBS, it appears to give greater representation and greater weight to the needs of the private sector. It is not obvious in the wording of the provisions how the AUSFTA will achieve the mooted benefits and avoid the possible risks to the PBS. This lack of clarity has generated considerable uncertainty and much criticism. The regulatory changes required by the AUSFTA, to strengthen intellectual property protection for example, could increase the reward to manufacturers for innovation, but could also substantially reduce government capacity to apply price controls in the pharmaceutical sector. Without the bargaining authority afforded by expert cost-effectiveness evaluation and reference pricing, government capacity to sustain historically desirable and medically/socially acceptable sector outcomes is far from certain. One hypothesis is that the AUSFTA may result in increased industry investment, but perhaps only at the cost of reduced equity of access.

## Framework for Evaluation of the AUSFTA's Medicines Impacts

Few studies have made direct measurements of the effects of trade agreements on access to medicines. The evidence for the putative benefits of stronger pharmaceutical patent rights – increased drug innovation, development of local drug research and development capacity and enhanced overall welfare of an introducing nation, is ambivalent. Some evidence suggests such strengthened intellectual property monopolies can stimulate invention, at least in encouraging incremental improvements by originators[35]. Other evidence suggests they freeze out future radical inventors[36]. Increased pharmaceutical intellectual property protection appears to have little positive impact on the level of local medicinal research and development[37]. A number of reasonably well-structured research projects conducted by competent scholars, in fact, have failed to find credible evidence that stronger intellectual property rights stimulates local pharmaceutical innovation[41]. Such rights, on the other hand, may substantially increase medicines prices in the countries that introduce it[38]. They can can also produce major changes in the local pharmaceutical industry that do not favour cheaper generic products[39]. This appears likely to produce major adverse health impacts for disadvantaged sectors of the population[40].

Overall, the welfare effects of global patent protection appear to be asymmetrical with the welfare in the inventing country rising with the extension of patent protection, while that of the introducing country falls by a proportionately greater amount[42]. The evidence rather suggests that the impact of strengthened intellectual property protection in developed nations such as Australia depends on the extent to which government regulation facilitates the continuance of generic pharmaceutical competition[43]. To what extent the viability of a nation's generic pharmaceutical industry should be resolved by the operation of market forces or lobbying from the brand name industry are major policy questions in this area[44].

While often not directly measurable, change in the character of Australian pharmaceutical regulation may be observable in how Australia's regulatory system materially and normatively responds to the changes required by the AUSFTA. We plan to observe the impact of the AUSFTA on key impact points of the regulation and governance of Australia's pharmaceutical sector and to track the effects of associated changes on drug expenditure, industry activity and medicine utilisation and affordability.

Many potential impacts are unlikely to be immediately observable; more probably it will be years before some changes are manifest. It is also probable that there will be sequelae, benign or otherwise, that are unable to be predicted. Our study, consequentially, will be ongoing with a wide focus, but provisional and responsive to what emerges as significant over time. Our broad interests – pharmacoeconomic, legal, public health, regulatory and socio-political – will be drawn on for relevant methodological and theoretical options for collecting and analysing qualitative and quantitative data from a range of primary and secondary sources.

Our plan on the quantitative side of the Project, is for the research to move from simply identifying *association *between the AUSFTA and changes in the price and supply of pharmaceuticals in Australia, to the more useful objective of assigning *causation*. The weakness in attempting to model alternative policy scenarios relates to the amount and quality of empiric data available and acceptance of the underlying assumptions. Emphasis, rather, will be on the ARC research scholar Andrew Searles constructing a theoretical framework for viewing the impact of the AUSFTA on the price and supply characteristics of pharmaceuticals in Australia drawing upon established economic theory, particularly in relation to public goods. We will also investigate the validity of the economic assumptions underpinning the AUSFTA medicines provisions. A pharmaceutical price index will be constructed taking into account the potential performance of its formulae under both axiomatic and economic approaches.

### Regulation and Governance

We aim to identify changes to regulatory structure and process, particularly the application of PBS reference pricing, associated with interpretation of the AUSFTA provisions. Material changes range from the possible – amendments to or repeal of relevant legislation; to the probable – changes to the processes and relationships within and between, the TGA, the PBAC (and its subcommittees) and the PBPA. We will also investigate the impact of the AUSFTA on the normative order applied in the deliberative processes. Changes, for example, might involve modifying the PBAC its evaluative process in ways that are mutually beneficial (transparency) or less so (reward of innovation taking precedence over cost-effectiveness). The extent to which impacts relates to core social justice principles such as equity of access to essential medicines will be a major focus, as mentioned, of the work of ARC research scholar Warwick Neville.

Quantitative and qualitative methods will be used longitudinally to observe for such changes and associated outcomes. These could include variation in the number of actual and possible listings, number of rejections of asking price (introductory price and price readjustments) and changes to the number of applications that include non-clinical claims concerning price. The number and type of proposals aired in the media by government and industry concerning pharmaceutical regulation will also be studied. The extent to which such proposals rely on adequate economic or other research, or other forms of justification, may also be investigated.

One hypothesis is that the AUSFTA may lead to changes in the type of medicines-related provisions included in subsequent bilateral trade agreements. Our research in this context will also explore the extent to which "innovation" and/or cost-effectiveness evaluation of new pharmaceuticals can be considered global public goods and what type of long term strategies can be developed to enhance their rational development. The project, for example, may consider the regulatory and fiscal advantages of bilateral trade deals, such as the China-Australia Free Trade Agreement, including a medicines cost-effectiveness working committee to facilitate exchange of pharmacoeconomic expertise[45]. This, or proposals for a multilateral treaty on the same topic, in part, may provide an economic and social justice balance to the potential impact of the AUSFTA on the PBS processes[46]. Creation of a medicines cost-effectiveness treaty, or related committees in bilateral trade agreements, could be a policy change that promotes quality use of medicines in all nations so involved[47]. Qualitative methods of the Project in this area will include key stakeholder interviews and a follow-up questionnaire.

### Industry Activity

We will examine the effects of the AUSFTA on the activity and returns of originator and generic manufacturers, including changes in profitability ratios, increases in employment and changes to Australia's pharmaceutical balance of trade. For originators, relevant indicators would include changes to monopoly rent for pharmaceutical patent holders; the number of applications to the TGA and PBS for listing of innovative patented products; changes to investment in research and development; and changes to expenditure on promotion and marketing. For generic manufacturers, we plan to observe for changes to the number of applications for marketing approval and changes to the timing of generic entry.

### Drug Expenditure

The Project will observe for changes in Federal and State government pharmaceutical expenditure associated with AUSFTA provisions – increased patent protection leading to delays in generic entry for example. This will include calculating the opportunity cost for other health areas of increased Federal and State hospital expenditure on innovative medicines. Direct and indirect (changes to over-the-counter drugs) price effects will be monitored. This may involve observing the pricing trends of strategically selected brand name products nearing patent expiration and the rate, price and number of relevant generic market entrants compared against expected results. As mentioned, for this outcome component the ARC research scholar Andrew Searles will develop an Australian pharmaceutical price index ('PPI') in an Excel spreadsheet, its design allowing the user to define subgroups of medicines.

### Drug Availability

We will examine whether AUSFTA required changes result in an increased availability of innovative drugs, faster access to subsidies for new prescription medicines and changes in the mix of generic and brand name drugs in the Australian market. This may also involve an independent expert evaluation of the innovative aspects of new drugs and post-marketing surveillance of associated treatment outcomes.

### Drug Utilisation and Affordability

We will observe for changes to overall drug utilisation and for changes to the use of newer innovative drugs compared to existing therapies on the PBS. We will also observe for changes to out-of pocket patient charges, for example increases in prescription co-payments, linked to the increased PBS expenditure associated with use of innovative medicines. Australian medicine users will be surveyed for changes to affordability of medicines following increases in out-of-pocket cost.

## Conclusion

As in other industrialised countries, regulation of the Australian pharmaceutical sector is an uneasy contingent system, its character reflecting the normative strength of private, relative to public, policy imperatives. With the recently implemented bilateral AUSFTA, however, the balance of such power may shift. The numerous material changes required by the AUSFTA in the pharmaceutical sector potentially aggregate to create a regulatory environment more attuned to encouraging private investment and profit-making. One hypothesis, in this context, is that the AUSFTA represents a normative shift that may dramatically change not only the character of Australian regulation but the protection of global public goods involved with assuring the general community obtains value for its pharmaceutical expenditure. It is uncertain to what degree a public health scheme with strong social justice traditions, like the PBS, can operate credibly under such changed conditions. The experience we detect is likely to be relevant to other countries with highly subsidised health care systems contemplating trade agreements with countries that have strong patent-protected pharmaceutical industries.

Plausibly the provisions of the AUSFTA represent minor procedural adjustments and leave the PBS intact. Some AUSFTA articles, however, clearly intend to create an expectation that distinct changes, to current Australian rather than US, pharmaceutical regulatory arrangements, will occur. Determining what alterations occur, and with what public health outcomes, will require careful empirical observation and theoretical analysis.

In summary, the potential exists for the AUSFTA to reshape the character of Australia's regulatory system concerning medicines- from a public good to a private rights-oriented system. Should the AUSFTA precipitate such a normative shift (particularly one away from scientific cost-effectiveness evaluation of pharmaceuticals) the regulatory implications are likely to be profound and resonate beyond Australia to impact on the health care sectors of other nations.

## Funding statement

'The Impact of International Trade Agreements on the Regulation and Provision of Medicines in Australia' is a three-year study funded by the Australia Research Council's Discovery Grant (project DP0556635), the Australian National University and the University of Newcastle. The Chief Investigators are Dr Thomas Faunce (Project Director), Professor Peter Drahos and Professor David Henry. Australian Research Council funded PhD scholars under the Project are Andrew Searles and Warwick Neville.

## Competing interests

The author(s) declare that they have no competing interests.
